# Is Insulin Resistance the Driver or the Outcome of Diabetes Mellitus? A Critical Review

**DOI:** 10.1155/jnme/9614195

**Published:** 2026-09-08

**Authors:** Shafa Shahid, Saadia Qamar, Fouzia Qamar, Aliza Fatima, Endre Máthé, Muniba Khaliq

**Affiliations:** ^1^ Department of Biotechnology, Faculty of Biological Sciences, Lahore University of Biological and Applied Sciences, Lahore 53400, Pakistan; ^2^ Biology Department, Faculty of Basic Sciences, Lahore Garrison University, Lahore, Pakistan, lgu.edu.pk; ^3^ Department of Food Science and Human Nutrition, University of Veterinary & Animal Sciences, Lahore 54000, Pakistan, uvas.edu.pk; ^4^ Faculty of Agricultural and Food Sciences and Environmental Management, Institute of Nutrition, University of Debrecen, Böszörményi Str. 128, Debrecen HU-4032, Hungary, unideb.hu; ^5^ Department of Life Sciences, Faculty of Medicine, Vasile Goldis Western University of Arad, L. Rebreanu Str. 86, Arad 310414, Romania, uvvg.ro; ^6^ Department of Food and Nutritional Sciences, University of Reading, Reading, Berkshire, UK, reading.ac.uk

**Keywords:** diabetes mellitus, hyperglycemia, insulin resistance, metabolic disorder, public health

## Abstract

Insulin resistance (IR) and Type 2 diabetes mellitus (DM) are intricately related metabolic conditions that significantly contribute to the global health burden. IR is characterized by chronic hyperglycemia that triggers a cascade of metabolic dysfunctions, including alterations in insulin signaling and the promotion of IR *via* inflammatory pathways. Concurrently, IR intensifies the progression of DM, resulting in a vicious cycle of metabolic decline and hyperglycemia. This review critically evaluates whether IR is a cause, an effect, or both in the pathogenesis of DM. This study seeks to clarify the causal relationship between IR and DM by integrating mechanistic insights, clinical data, and recent epidemiological studies. However, emerging evidence suggests that IR can be both a cause and an effect in DM, depending on the stage and type of the disease. Public health implications play an important part in the prevention of DM, particularly in developing regions where the prevalence of DM is escalating. Early detection of IR, lifestyle interventions, and therapeutic strategies, coupled with digital health initiatives and urban design, present unique avenues for addressing this worldwide health challenge. Future research must address these gaps in causal inference by undertaking long‐term studies, adopting standardized definitions, and implementing precision medicine applications *via* multiomics approaches. Recognition of IR as both a driver and consequence of diabetes emphasizes the urgent necessity for targeted and comprehensive strategies that address its progression and root causes across different populations.

**Highlights**
• This review emphasizes the fact that insulin resistance can act as both cause and consequence of disease progression in both T1DM and T2DM.
• Experimental models and epidemiological data confirm the self‐perpetuating cycle between IR and hyperglycemia, reinforcing the need for early detection of IR and hyperglycemia by using validated biomarkers and techniques.
• Breaking the vicious cycle of IR and diabetes at multiple mechanistic, clinical, and societal levels is crucial for curing the worldwide diabetes epidemic.

## 1. Introduction

Diabetes mellitus (DM) is a multifactorial metabolic disorder that is characterized by chronic hyperglycemia due to unwanted alterations in insulin action. Diabetes‐related death rates had increased by 3% from 2000 to 2019. This rate corresponds to 13% in lower‐ to middle‐income countries. According to the International Diabetes Federation, around 537 million adults were found to be victims of diabetes in 2021 globally, and this number has dramatically increased in recent years [1].

DM is mainly classified into two types: Type 1 DM (T1DM) and Type 2 DM (T2DM). T1DM is an autoimmune condition that can destroy pancreatic β‐cells, resulting in an absolute deficiency of insulin. It accounts for 5% to 10% of all diabetic cases worldwide. However, T2DM accounts for over 90% of overall worldwide cases and is characterized by a progressive decline in insulin production and insulin resistance (IR). T2DM normally appears in later life, but due to an increase in obesity, lack of physical activity, and bad eating habits, it is increasingly prevalent in younger individuals [2]. Insulin is a key anabolic hormone that regulates the metabolism of glucose, protein, and lipids. If not managed properly, uncontrolled diabetes can result in several complications, including confusion, coma, and, in rare cases, death because of nonketotic hyperosmolar (hyperosmolar hyperglycemic state [HHS]) syndrome.

All tissues with insulin receptors can become insulin resistant, but the tissues that primarily drive IR are the liver, skeletal muscle, and adipose tissue. IR is a condition that occurs when normal or elevated insulin levels produce a weak biological response, typically resulting in impaired sensitivity to insulin‐mediated glucose elimination, which includes utilization of glucose in peripheral tissues, inhibition of lipolysis in adipose tissue, and suppression of hepatic glucose production [3]. In order to compensate for this loss, the pancreas produces more insulin, thus leading to the state of hyperinsulinemia [4]. This compensation declines over time, leading to an elevation in glucose levels and the eventual development of T2DM. IR is a key feature of metabolic syndrome that includes several conditions, such as hypertension, dyslipidemia, obesity, impaired glucose tolerance, elevated inflammatory markers, endothelial dysfunction, and a prothrombotic state [5]. These interrelated factors significantly enhance the risk of T2DM and heart disease, emphasizing the importance of IR in overall metabolic health [6]. The link between IR and DM is complicated and bidirectional. In T2DM, IR is widely acknowledged as a main causative factor, often occurring before the onset of hyperglycemia, particularly when combined with β‐cell dysfunction. However, studies have shown that DM can impair insulin signaling, implying that IR may also develop or be elevated because of diabetes. Autoimmune destruction of pancreatic β‐cells is the main cause of T1DM; IR can occur later due to various factors like obesity, puberty, complicating disease management, or long‐term insulin therapy. Consequently, current findings support that IR can be both the cause and effect of DM, depending on the stage and type of the disease [7].

### 1.1. Problem Statement

Whether IR operates as a precursor to diabetes or as a downstream consequence of chronic hyperglycemia, the point at which the causality reverses, still remains undefined. This ambiguity has given rise to a translational gap: Prevention strategies advocate that IR should be targeted early on as a priority, while management strategies of established diabetes pronounce IR as a secondary phenomenon. On account of this, the literature and clinical guidelines are lacking explicit criteria or a unified framework to distinguish the two states in practice. There is also a scarcity of consolidated evidence on how disease stage, type, and population‐specific factors affect this relationship, thereby leading to inconsistent interpretations in both research and clinical practice. Current evidence underscores a critical need for a comprehensive review of this relationship. Existing clinical management often focuses on glycemic control after diabetes onset rather than early detection and intervention at the IR stage. Subsequently, the risk prediction models, screening programs, and therapeutic trials will end up in false intervention mismatching the actual pathophysiology at a given disease stage.

### 1.2. Objectives

Considering the identified research gaps, the current review aims to synthesize and critically analyze the latest scientific findings on pathophysiological interplay between IR and DM. A major focus will be on delineating the mechanisms linking IR and DM, and how the integration of mechanistic, clinical, and epidemiological evidence can clarify this causal shift to provide a comprehensive overview of their bidirectional interplay.

This review also intends to highlight preventive approaches and public health strategies to avoid or delay the onset of the disease, which may prove especially significant for at‐risk populations living in developing countries, where the prevalence of diabetes is accelerating at a rapid pace.

## 2. Materials and Methods

The current narrative review is designed to draft a comprehensive report on the bidirectional relationship between IR and DM. Major scientific databases were selected for comprehensive searches, including Google Scholar, PubMed, and Scopus, from the last ten years (2016 to 2026). Considering the identified research gaps, the literature was compiled by searching original research articles, clinical trials, and high‐impact review articles that provide detailed insight into mechanistic, clinical, epidemiological, and interventional findings related to the topic of the intended narrative review article. Key search concepts included “insulin resistance,” “type 2 diabetes mellitus,” “type 1 diabetes,” “causality,” “beta‐cell dysfunction,” “insulin sensitivity,” “insulin signaling,” “hyperglycemia,” “glucose homeostasis,” “epidemiology,” “diabetes mellitus and insulin resistance bidirectional relationship,” “mechanistic pathways,” “management of insulin resistance,” and “preventive and therapeutic strategies targeting insulin resistance.”

## 3. Etiology of IR

IR may also exist as a regulated component of metabolic homeostasis. In healthy individuals, reversible and tissue‐specific reduction in insulin sensitivity can be observed under certain physiological conditions like stress, growth, and nutritional scarcity. It can work as a metabolic traffic controller and divert glucose and other nutrients to the physiologically prioritized tissues. During fasting, the transient state of IR is induced in the peripheral tissues, leading to fatty acid consumption and preserving glucose for tissues with obligatory glucose requirements like the brain. Similarly, during pregnancy, the secretion of placental hormones facilitates the availability of glucose to the developing fetus by inducing IR in maternal skeletal muscles [8, 9]. However, the persistent disruption of feedback networks due to chronic nutrient overload can divert the pathway to a permanent pathological IR state [6, 10]. IR arises due to complex interactions between lifestyle and environmental factors and genetic predisposition. Both forms contribute to the impairment of normal insulin signaling, thereby resulting in disruption of glucose homeostasis and progression toward metabolic diseases such as T2DM [11].

### 3.1. Genetic Etiology

Genetic factors play an important part in influencing an individual’s susceptibility to IR. According to genome‐wide association studies (GWAS), multiple loci are associated with insulin sensitivity and β‐cell function. Variants in genes including PPARG, insulin receptor substrate‐1 (IRS‐1), TCF7L2, and FTO have been implicated in altered insulin receptor signaling, adipocyte differentiation, and lipid metabolism [12]. Furthermore, monogenic syndromes, such as mitochondrial disorders, insulin receptor gene mutations, and lipodystrophies, may lead to IR independent of lifestyle factors or obesity. The genetic anomalies often impair the action of insulin at the receptor or postreceptor level, which results in the early onset of metabolic disorders [13].

### 3.2. Acquired Etiology

Acquired IR develops due to various modifiable risk factors such as high‐calorie diets, sleep deprivation, obesity, chronic inflammation, sedentary behavior, and aging. Obesity plays a vital role in the progression of IR, as visceral adipose tissue secretes proinflammatory cytokines, including IL‐6 and TNF‐α, which interfere with the phosphorylation of IRS and downstream signaling. Chronic excess of nutrients also results in glucotoxicity and lipotoxicity, thereby impairing the action of insulin in adipose tissue, liver, and muscle. Other acquired contributors include certain drugs (e.g., antipsychotics and corticosteroids), endocrine disorders (e.g., polycystic ovarian syndrome [PCOS] and Cushing’s syndrome), and stress‐related hormonal imbalances, all of which can disrupt insulin sensitivity [14].

## 4. Insulin Signaling and IR

### 4.1. Normal Signaling Pathway of Insulin

The insulin signaling pathway is a central regulator in glucose metabolism and overall energy homeostasis. Under normal conditions, insulin is secreted from pancreatic β‐cells in response to increased blood glucose levels, particularly after food intake. Insulin binds to the insulin receptor, which is present on the surface of insulin‐responsive cells, such as adipose tissue, muscles, and the liver. Following this, it undergoes autophosphorylation at the tyrosine residues present at intracellular β subunits, triggering an intracellular signaling cascade.

**FIGURE 1:**
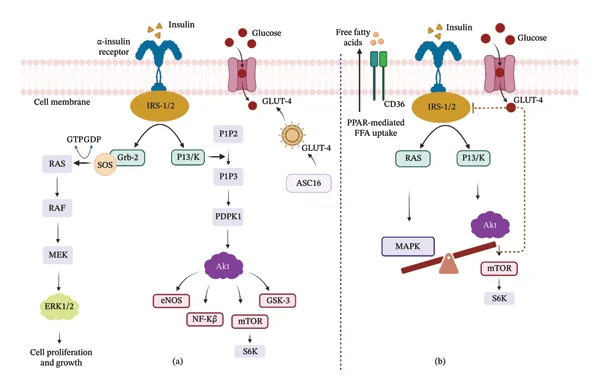
Insulin signaling pathway (a) normal state of insulin signaling pathway, (b) insulin resistance state. Created in BioRender https://BioRender.com/2cayi6g.

IRS proteins, particularly IRS‐1 and IRS‐2, undergo phosphorylation after the activation of the receptor and function as key adapter molecules. These phosphorylated IRS proteins activate and interact with phosphatidyl‐inositol 3‐kinase (PI3K), which results in the formation of phosphatidyl‐inositol‐3,4,5‐triphosphate (PIP3). PIP3 serves as a docking site for protein kinase B (Akt) and PDK1, which is its upstream activator. After complete activation, Akt stimulates the translocation of glucose transporter Type 4 (GLUT4) to adipose tissue and skeletal muscle plasma membrane, thus allowing uptake of glucose into the cells from the bloodstream via inhibition of glycogen synthase kinase 3 (GSK‐3). Activated Akt also stimulates the mTOR pathway by inhibiting the TSC1/TSC2 complex, consequently activating mTORC1, which in turn promotes lipid/protein biosynthesis and glucose consumption through downstream regulation of factors like S6 kinase 1 and 4E‐BP1, sustaining cell growth and metabolic homeostasis. Simultaneously, insulin triggers the Ras/MAPK pathway through the Grb2–SOS complex, which is primarily associated with gene expression and cell proliferation rather than metabolic regulation (Figure 1) [15, 16].

### 4.2. Insulin Signaling in Case of IR

Metabolic roots of acquired IR lie in the disruption of insulin signaling by downregulating the glucose‐transport system. In the IR state, body cells fail to effectively respond to normal or even increased insulin levels, leading to disrupted glucose metabolism and hence absorption. Attenuated phosphorylation of IRS proteins is one of the earliest defects caused by persistent exposure to inflammatory cytokines like TNF‐α, oxidative stress, or accumulation of lipids. These stressors induce threonine/serine phosphorylation of IRS proteins, which inhibits the activation of PI3K, thereby disrupting the signaling cascade of Akt. As a result, translocation of GLUT4 is reduced, thus inhibiting the uptake of glucose in peripheral tissues, whereas the MAPK pathway is preserved, which may result in vascular injury in endothelial cells by the mitogenic‐promoting effect (Figure 1). The chronic hyperglycemic state in blood plasma and body tissues is an indicator of reduced expression and translocation of GLUT4. This is not a primary genetic defect in most cases, but a responsive downregulation to the overabundance of substrate [17].

IR in the liver leads to continuous production of hepatic glucose, despite high levels of circulating insulin, thereby contributing to hyperglycemia. Furthermore, impaired activity of Akt leads to reduced inhibition of gluconeogenic enzymes [18]. In adipose tissues, IR promotes lipolysis and reduces lipogenesis, thus elevating levels of free fatty acids that further aggravate systemic IR. Chronic IR plays a critical role in the development of several metabolic disorders and elevates the demand for pancreatic β‐cells to produce more insulin, which leads to compensatory hyperinsulinemia, which eventually leads to dysfunction and depletion of β‐cells [19]. The NMR spectroscopy using phosphorus and carbon isotopes revealed that the muscle glycogen synthesis is severely impaired, which is the most important pathway of glucose utilization. Surprisingly, the studies show that the offspring of diabetic patients demonstrate some impairments in glycogen synthesis in early life, but once the hyperglycemia is established, the IR escalates at an exponential rate.

In a chronic excess nutrient state, when hyperinsulinemia or inflammation occurs, sustained mTORC1 activation leads to a negative feedback loop, which causes IR. Namely, the S6K1 phosphorylates IRS‐1 on inhibitory serine residues, as a result of which IRS‐1 is unable to mediate insulin receptor transmission. This negative feedback mechanism attenuates PI3K/Akt signaling, leading to reduced glucose uptake and impaired metabolic regulation. Consequently, chronic overactivation of mTOR signaling contributes to the development of IR, a key factor in the pathogenesis of metabolic disorders such as T2DM [19].

However, the transient nature of physiologically induced IR in healthy individuals is largely attributable to the reversible alteration in the insulin signaling cascade regulation. Under physiological stimulus, the core signaling machinery remains structurally intact, so once the stimulus is removed, the insulin signaling network gets restored. It typically involves attenuation of insulin signaling through insulin receptor, IRS, and PI3K/Akt pathway. Kinases phosphorylate specific regulatory serine residues. Once the insulin level reduces, phosphatases dephosphorylate these residues, resulting in the restoration of IRS‐1 back to normal [10, 20, 21].

## 5. IR: A Predecessor or Successor

### 5.1. IR as a Precursor of T2DM

#### 5.1.1. Epidemiological Evidence

Recent extensive epidemiological evidence has elaborated that IR is a major risk factor in the development of T2DM, especially among prediabetic individuals and those presenting with metabolic syndrome. Prediabetes is a clinically recognized intermediate state that is the consequence of impaired glucose tolerance and impaired fasting glucose, which represents early anomalies in β‐cell function and insulin sensitivity. According to longitudinal cohort research on the Atherosclerosis Risk in Communities (ARIC) and Framingham Offspring study, individuals with higher baseline IR are more likely to develop T2DM, as indicated by surrogate markers such as the homeostatic model of IR (HOMA‐IR) or fasting levels of insulin [22].

Furthermore, metabolic syndrome, defined by a set of risk factors such as hypertriglyceridemia, obesity, low HDL, hypertension, and elevated fasting glucose levels, is reported to be strongly associated with reduced insulin sensitivity. Data from DECODE and NHANES indicate that those individuals who meet the criteria of metabolic syndrome have a 2–5‐fold increased risk of developing T2DM, independent of their family history, age, and ethnicity [23]. Additionally, large trials, including the Diabetes Prevention Program (DPP) and the Finnish Diabetes Prevention Study (DPS), have shown that insulin sensitivity can be improved through lifestyle changes or can postpone T2DM through metformin [24].

In 2023, a study involving data from over 78,000 Chinese adults showed that cumulative exposure to metabolic risk factors, such as an increase in fasting plasma glucose, triglycerides, blood pressure, and body mass index, led to increased risk of developing T2DM. Individuals with a high cumulative metabolic score for IR (MetS‐IR) had an odds ratio of diabetes progression in [25]. Similarly, in 2024, a cohort study involving about China’s 15,423 prediabetic adults, researchers found that an increased MetS‐IR was linked to a reduction in glycemic reversion by 3.2% and 3.7% in females and males, respectively [26].

#### 5.1.2. β‐Cell Compensation and Exhaustion

During the initial stages of IR, pancreatic β‐cells enhance secretion of insulin to sustain normal blood glucose levels; this process is known as β‐cell compensation. Initially, compensatory hyperinsulinemia effectively compensates for reduced insulin sensitivity in peripheral tissues. Recent research has shown that hyperactivity of β‐cells for a long time results in endoplasmic reticulum stress, disrupted function of mitochondria, and oxidative damage, which disrupts the synthesis of insulin and leads to insulin insufficiency over time [27].

**FIGURE 2:**
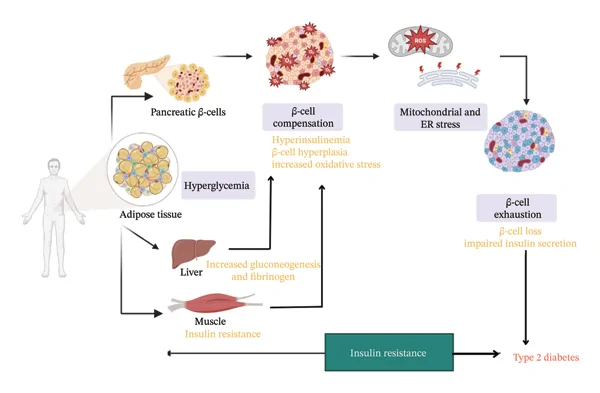
Process of β‐cell compensation and exhaustion leading to Type 2 diabetes. Created in BioRender https://BioRender.com/alojb1j.

IR results in progressive degradation of β‐cell function, ultimately leading to β‐cell exhaustion, which is a state in which the output of insulin is insufficient to overcome peripheral resistance (Figure 2). Longitudinal studies, such as the United Kingdom Prospective Diabetes Study (UKPDS), reported that decline in activity of β‐cell is a crucial factor in the progression from prediabetes to the T2DM state. Moreover, pancreatic islet histopathology in T2DM patients showed a reduction in the mass of β‐cells, imparted by reduced regeneration and apoptosis, rather than dysfunction alone [28]. This progressive failure of β‐cells is intensified by glucotoxicity and lipotoxicity, resulting from increased circulating free fatty acids and sustained hyperglycemia, respectively [27, 29].

### 5.2. DM as a Contributor to IR

#### 5.2.1. Glucose‐Induced IR

In the context of DM, chronic hyperglycemia not only indicates underlying IR but also acts as a pathogenic factor that exacerbates insulin sensitivity; this condition is known as glucose‐induced IR. High glucose levels also stimulate the biosynthesis process of hexosamine, resulting in the accumulation of O‐linked *N*‐acetylglucosamine (O‐GlcNAc) alterations on insulin signaling proteins, further disrupting their function [30]. DM causes the release of proinflammatory cytokines such as IL‐6 and TNF‐α, thus leading to the activation of isoforms of protein kinase C (PKC) and increased flux through advanced glycation end‐product (AGE) pathways and the polyol pathway. All these factors contribute to IR by constraining downstream signaling efficacy and insulin receptor sensitivity. Moreover, chronic hyperglycemia impairs the balance of adipokines and reduces the level of adiponectin, thereby diminishing uptake of glucose in adipose and muscle tissues. These outcomes create a self‐perpetuating cycle, where disrupted signaling of insulin exacerbates hyperglycemia, which in turn worsens IR. In this setting, progression of diabetes by IR emphasizes the bidirectional relationship between defective action of insulin and hyperglycemia [6].

#### 5.2.2. AGEs and Cellular Damage

In a prolonged hyperglycemic state, the nonenzymatic glycation between reducing sugars and biomolecules like protein, lipid, and nucleic acid results in the production of AGEs. Since these AGEs characterize the chemical environment of diabetes, therefore the AGEs are no longer observed as an indicator of hyperglycemia but as potent inhibitors of insulin signaling [31].

The pathway of AGE biosynthesis begins with the condensation of glucose with the amino group of proteins to form Schiff bases that eventually rearrange into stable Amadori intermediates. These intermediates further undergo dehydration and oxidation reactions to form AGE products such as methylglyoxal‐derived AGEs, Nɛ‐(carboxymethyl)lysine, and pentosidine. Furthermore, the occurrence of oxidative stress during diabetes amplifies the formation of AGEs via glycoxidation processes. The accumulation of AGEs causes them to crosslink structural proteins, including collagen, impairing elasticity and matrix turnover in tissues, thus leading to reduced cellular integrity and stiffness.

**FIGURE 3:**
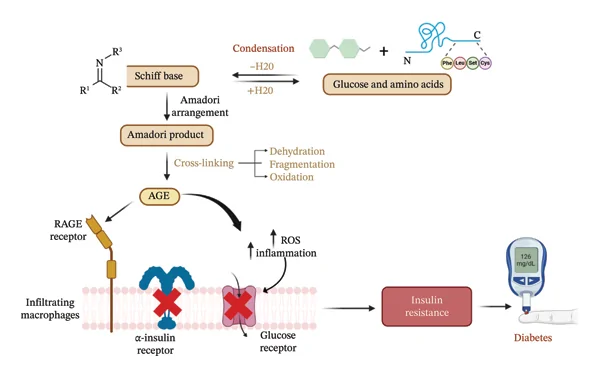
Disruption of insulin signaling by AGE. Created in BioRender https://BioRender.com/17o3c2z.

AGEs bind the receptor for advanced glycation end‐products (RAGEs) and initiate a “feed‐forward‐fueled pathological loop.” This interaction induces the activation of Ras‐mitogen‐activated protein kinase and the NF‐kB pathways, triggering macrophage infiltration and production of proinflammatory cytokines. The cytokines upregulate the serine phosphorylation of insulin receptors, casting an inhibitory effect on the insulin signaling cascade. These effects are compounded in the liver, in which AGEs enhance hepatic gluconeogenesis and result in high fasting glucose levels (Figure 3) [32].

In addition to disrupting the insulin signaling pathways, AGEs can directly modify the insulin molecule, thereby reducing glucose uptake by the cell. The glycation of the beta chain of the insulin molecule at the phenylalanine position can lower the efficiency of the insulin peptide by 20%. Moreover, a significant AGE precursor accumulates in the pancreas during the diabetic state and is able to modify the insulin and proinsulin molecules into a biologically less active form. Such circumstances lead to the creation of a state of IR in which the signaling molecule is functionally compromised, instead of a defect in body tissue.

#### 5.2.3. Ectopic Fat and Lipotoxicity

Although IR is considered one of the vital reasons for obesity, there is sufficient evidence from metabolic signaling pathways that robustly claims that obesity causes resistance by lipid dysregulation. Once the subcutaneous adipose tissue reaches its threshold limit to store the lipid molecules, the excess fat is directed toward the nonadipose tissues, such as skeletal muscles, liver, and heart. The effect is called the lipid spillover effect.

In the context of incipient diabetes and nutrient overload, the accumulated ectopic fat serves as the harbor of toxic intermediate lipid metabolites, such as diacylglycerol (DAG) and ceramides. For instance, the accumulation of DAG in hepatic and myocellular tissues suppresses insulin signaling at the IRS protein level. This process usually occurs due to nutritional overload, which results in more insulin production, and insulin itself is a promoter of lipogenesis.

#### 5.2.4. Autoimmune Inflammation in T1DM

**FIGURE 4:**
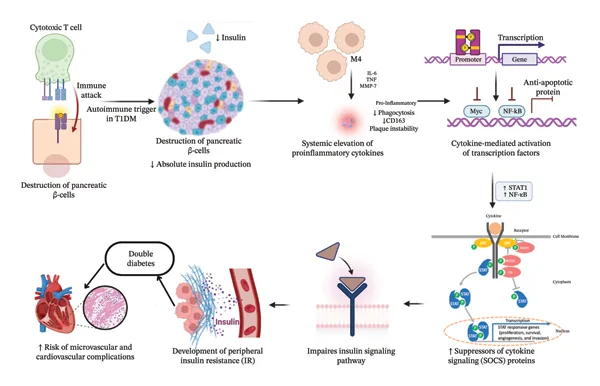
Cytokine‐induced IR in T1DM leading to emergence of phenotype of double diabetes. Created in BioRender https://BioRender.com/dx013kw.

T1DM involves autoimmune‐mediated destruction of pancreatic β‐cells, resulting in absolute insufficiency of insulin. Research indicates that the disease process is not only limited to failure of β‐cells; it is also associated with cytokine‐induced IR in peripheral tissues. Exposure of cytokines activates proinflammatory transcription factors such as STAT1 and NF‐κB, leading to the activation of genes that inhibit the signaling of insulin [33]. Furthermore, suppressors of cytokine signaling (SOCS) proteins disrupt the function of the insulin receptor and promote degradation of IRS. Elevated circulating cytokines have been linked to reduced insulin sensitivity in both human and animal models, even in patients receiving exogenous insulin. These findings explain why individuals who are overweight and in their puberty develop a phenotype of “double diabetes,” thereby exhibiting both autoimmunity and IR. Double diabetes involves a dual burden of IR and autoimmune diabetes that complicates disease management and increases the risk of microvascular and cardiovascular complications (Figure 4) [34].

#### 5.2.5. Exogenous Insulin Therapy and Resistance in T1DM

Exogenous insulin therapy is essential for glycemic control in T1DM to prevent autoimmune destruction of the pancreatic β‐cell. However, recent research suggests that in the case of suboptimal glycemic regulation, persistent exposure to high doses of exogenous insulin may contribute to peripheral IR in overweight individuals with T1DM. In contrast, endogenous insulin that first acts on the liver and is directly secreted into portal circulation, and subcutaneously administered insulin causes nonphysiological peripheral hyperinsulinemia, thus exposing adipose tissue and skeletal muscle to disproportionately increased levels of insulin [35].

Downregulation of expression of the insulin receptor may occur due to prolonged hyperinsulinemia that can impair phosphorylation of IRS and disrupt downstream insulin signaling, all of which contribute to postreceptor defect in action of insulin [36]. Furthermore, studies have indicated that increased use of exogenous insulin to prevent poor glycemic control and IR may lead to adipogenesis, thereby promoting exacerbation of insulin sensitivity and increased fat mass. This phenomenon is known as the insulin resistance obesity cycle [37].

## 6. A Bidirectional Relationship: Cause and Consequence

### 6.1. Vicious Cycle Between Hyperglycemia and IR

The evidence accumulated above suggests that the relationship between IR and hyperglycemia in the pathophysiology of T2DM is nonlinear but is inextricably linked through a self‐reinforcing cycle. This bidirectional relationship suggests that IR results in elevated blood glucose levels, whereas chronic hyperglycemia worsens IR. Hyperglycemia also alters the gene expression related to insulin sensitivity and contributes to lipotoxicity in combination with elevated levels of free fatty acids. These metabolic disruptions impede the translocation of GLUT4 transporters, promote the production of hepatic glucose, and reduce the uptake of glucose by peripheral tissues, thereby making IR worse and reinforcing hyperglycemia [38]. The toxic intracellular environment due to the accumulation of abundant glucose, AGEs, and ectopic fats unequivocally leads to IR. This resistance acts as a precursor to worsening the situation further by creating a pathway of metabolic decay called a vicious cycle.

**FIGURE 5:**
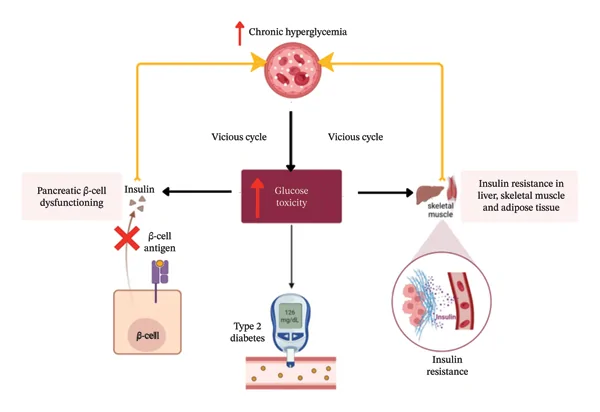
Vicious cycle of insulin resistance and Type 2 diabetes mellitus. Created in BioRender https://BioRender.com/5a8zagz.

This interconnected progression results in the creation of a metabolic vicious cycle, in which both IR and hyperglycemia cannot properly work without targeting the other (Figure 5). This bidirectional relationship has been substantiated in both experimental and clinical studies, thereby demonstrating that hyperglycemia is not solely a consequence but also a driver of IR. The longer this cycle gets, the larger the risk of irreversible exhaustion of the β‐cell. To effectively manage the condition, it is crucial to break this cycle early through lifestyle interventions, glucose‐lowering therapies, and insulin sensitizers, which can help halt disease progression and restore metabolic balance [39].

### 6.2. Evidence From Animal and Human Models

A study was conducted in 2023, where dogs were fed a high‐fructose and high‐fat diet for several weeks. This resulted in systemic IR, impaired function of beta cells, and decreased tolerance to glucose, thereby closely mimicking the initial stages of human T2DM development. This study demonstrated that IR precedes hyperglycemia, and diet‐induced IR can impair β‐cell function, accelerating diabetes progression in a human‐applicable model [40]. Another animal model study conducted previously in 2021 involving a novel mouse model was proposed, combining dexamethasone, a glucocorticoid, a high‐fat diet, and low‐dose streptozotocin. The researchers observed that this multifactorial model stimulated both β‐cell failure and interdependence of IR witnessed in T2DM. High‐fat diet induced IR in muscle and adipose tissue, whereas streptozotocin damaged the β‐cell selectively, and dexamethasone disrupts insulin’s action by promoting hepatic gluconeogenesis [41].

A link between T2DM and muscle strength was established during a study that engaged the Mendelian randomization method. Genetically, lower handgrip strength has been linked to a higher risk of T2DM and subsequent muscle function decline. Since skeletal muscle is the primary site of disposal of glucose, this decreased muscle strength reflects insulin function impairment. This approach eradicates confounding, rendering this robust model for justifying the bidirectional relationship between T2DM and IR [42]. Research on liver‐specific insulin receptor knockout (LIRKO) mice has demonstrated that hepatic IR results in uncontrolled gluconeogenesis, systemic metabolic dysfunction, and hypertension [43].

## 7. Clinical Implications and Management of IR

### 7.1. Management of Insulin Resistance

The management of IR relies on a combination of pharmacological interventions, lifestyle modifications, and treatment of underlying risk factors. Evidence‐based guidelines highlight significant dietary modifications, notably high‐fiber diets, low‐glycemic index, and reduction in saturated fat intake, as they enhance insulin sensitivity and lower fasting glucose levels. Physical activity, particularly a combination of aerobic and resistance exercises, boosts insulin‐mediated uptake of glucose in skeletal muscle, which is the primary site of glucose disposal [44]. Weight loss as modest as 5%–10% of total body weight has a major role in the reduction of IR and associated cardiometabolic risks [45]. Management strategies also include treatment of comorbid conditions like dyslipidemia, hypertension, and sleep disorders, which may exacerbate IR. Additionally, individualized approaches, such as patient education and behavioral counseling, are crucial for effective outcomes [5].

### 7.2. Early Detection and Intervention of IR

Recognizing IR at its early stages provides a unique opportunity for timely intervention, thereby allowing the prevention of irreversible metabolic disruption and protecting overall health. The Whitehall II cohort study and DPP demonstrated that individuals with reduced insulin sensitivity often remained normoglycemic for prolonged periods but eventually transitioned to T2DM due to the cumulative impact of lipotoxicity, glucotoxicity, and β‐cell stress. These findings highlight that IR is not only an early warning sign but also a risk factor whose timely detection can delay or prevent the onset of disease [46]. From a clinical point of view, early detection of IR enables risk stratification in asymptomatic individuals, specifically in those with a sedentary lifestyle, obesity, PCOS, or a family history of diabetes. Measures of IR have not been incorporated into clinical guidelines. From a public health perspective, early detection promotes cost‐effective prevention strategies by lowering long‐term healthcare burdens associated with diabetes‐related complications. As a result, integration of IR screening into routine clinical assessments, particularly in populations with a high risk of IR, is highly recommended by experts to facilitate effective and timely interventions.

In 2009, a collaborative scientific statement unifying criteria for MetS was released. Identification of MetS is done by the presence of 3 or more of the following diagnostic cut points. Based on gender and ethnicity, waist circumference should be between 32” and 40”. For treating hypertriglyceridemia, elevated triglyceride levels should be greater than or equal to 150 mg/dL, or medication should be taken. HDL levels should be less than 40 mg/dL in males or less than 50 mg/dL in females. Blood pressure should be greater than or equal to 130 mm Hg systolic or greater than or equal to 85 mm Hg diastolic, or antihypertensive medication should be taken [5].

The American College of Endocrinology identifies specific physiologic abnormalities that increase IRS risk. These abnormalities include impaired tolerance of glucose or impaired fasting glucose, abnormal metabolism of uric acid, dyslipidemia (elevated triglyceride level, lower levels of HDL‐C, and dense LDL), elevated blood pressure, prothrombotic factors (PAI‐1 and fibrinogen), inflammation markers (C‐reactive protein and white blood cell count), and endothelial dysfunction. Some other factors may include the following: BMI should be greater than or equal to 25 kg/m^2^; diagnosis of CVD, PCOS, nonalcoholic fatty liver disease (NAFLD), or acanthosis nigricans; family history of T2DM, hypertension, or CVD; sedentary lifestyle; underrepresented ethnic groups; and age older than 40 years [47].

The American Diabetes Association evaluates individuals at high risk even after their fasting glucose level is normal. Tests like the triglyceride‐glucose (TyG) index or the HOMA‐IR can detect metabolic imbalance before hyperglycemia occurs [48]. Furthermore, early IR is not only linked to the risk of diabetes but also mechanistically associated with hypertension, reproductive dysfunction, atherosclerosis, and NAFLD. Proactive detection of IR enables targeted and timely interventions that preserve metabolic function and provide protection against a wide range of chronic diseases [49].

### 7.3. Emerging Diagnostic Molecular Biomarkers

Traditional markers such as HOMA‐IR, fasting glucose, insulin levels, and the glucose clamp technique provide useful but not exact estimates of insulin sensitivity; therefore, many emerging biomarkers offer precise insights into the underlying pathophysiology. Adiponectin is one of the most studied adipokines that enhances the sensitivity of insulin via activation of AMPK and the anti‐inflammatory pathway [50]. According to research, miRNAs have emerged as stable and sensitive biomarkers for IR, detectable in serum and plasma samples. miR‐29, miR‐126, and miR‐375 are strongly linked to glucose tolerance, inhibition of insulin signaling, and dysfunction of β‐cells in both preclinical and clinical studies [51].

Ceramides and acylcarnitines are reliable metabolic biomarkers that detect incomplete oxidation of fatty acids and lipotoxic stress, both of which disrupt insulin sensitivity [52]. Angiopoietin‐like protein 4 (ANGPTL4), complement component C3, and fibroblast growth factor 21 (FGF21) are well‐known proteomic biomarkers that are involved in modulating hepatic insulin sensitivity [53, 54].

### 7.4. Therapeutic Strategies Targeting IR

i. Lifestyle Interventions: Lifestyle modification is still one of the most effective strategies for reversing and preventing IR. Daily physical exercise plays an important part in improving insulin sensitivity by reducing visceral adiposity and uptake of glucose in skeletal muscles. Studies like the DPP Outcome Study (2023) and the AHEAD study (2021) indicated that weekly moderate‐intensity exercise for 150 min substantially reduces insulin levels and improves scores of HOMA‐IR [45]. By adopting a low‐glycemic index diet, lipid metabolism can be improved, and there can be an improvement in the action of insulin. In overweight individuals, weight loss of 5%–10% of body weight is associated with significant improvement in insulin sensitivity [55].
ii. Pharmacological Therapies: Metformin is a cornerstone treatment because of its ability to suppress the production of hepatic glucose and enhance the signaling of insulin via activation of AMPK. It is most recommended for patients with prediabetes and metabolic syndrome [56]. SGLT2 inhibitors, such as dapagliflozin and empagliflozin, are primarily glucose‐lowering agents but indirectly play an important role in improving IR by reducing adipose inflammation and reducing glucotoxicity [57]. Thiazolidinedione, which is a low‐dose pioglitazone, has reemerged as a viable choice in recent trials reporting improved action of insulin [58] (Table 1).
iii. Other Considerations:
  1. Sleep Quality: Poor sleep can contribute to IR; good sleep hygiene practices must be prioritized [63].
  2. Monitoring and Follow‐up: Regular tracking of blood glucose, insulin levels, and other health markers to adjust management strategies is required [64].
iv. Emerging Therapies:
  1. Nutraceuticals: Certain supplements like berberine, chromium, and omega‐3 fatty acids have beneficial effects on insulin sensitivity [65].
  2. Gut Microbiome Modulation: Research suggests that gut microbiome alterations may play a role in IR; probiotics and prebiotics may be beneficial [66].
  3. Cellular Replacement Therapies: Use whole cells or stem cell–derived cells to reverse IR or support insulin function. These are meant to replace dysfunctional cells or modulate the immune/metabolic environment (Table 2).
  4. Subcellular Particle‐Based Therapies: Nanoparticles, exosomes, or other cell‐derived vesicles are recruited to deliver drugs or signals for improving insulin sensitivity (Table 2).

**TABLE 1:** Medicinal and pharmacological therapies targeting insulin resistance (IR).

Medicine/agent	Mechanism of action	Effect on IR	Evidence from recent studies	References
Metformin	Enhances peripheral uptake, reduces hepatic gluconeogenesis, and activates the AMPK pathway	↑Insulin sensitivity, ↓HOMA‐IR, Enhanced Matsuda Index in individuals with hepatic IR.	MARCH trial subanalysis: subjects with high baseline hepatic insulin resistance derived the greatest improvements in HOMA‐IR and Matsuda Index after 48 weeks.	[59]
Hydroxychloroquine (HCQ)	Modulates cytokine signaling and insulin degradation pathways	Evidence of increased insulin sensitivity in PCOS and IR conditions	Systematic review (2021–2022) shows reductions in fasting insulin and HOMA‐IR	[60]
INF200 (NLRP3 inhibitor)	Blocks inflammasome‐mediated metaflammation	Preclinical evidence suggests restored insulin sensitivity in adipose‐inflammatory state	Obese rat model: improved glucose/lipid profiles with decreased inflammation	[61]
Tirzepatide	Dual GLP‐1 & GIP receptor agonist	↓ HOMA2‐IR by 15.5%–24.0% vs semaglutide’s ∼5%; improved insulin sensitivity beyond weight loss	Post hoc analysis of SURPASS‐2: at Week 40, greater HOMA2‐IR reduction across all tirzepatide doses vs semaglutide	[62]

**TABLE 2:** Cellular replacement and subcellular particle‐based therapies.

Approach	Therapeutic mechanism/s	Status	References
*(A) Cellular replacement therapies*			
Mesenchymal stem cell (MSC) therapy	These cells are multipotent stromal cells originating from bone marrow, adipose tissue, umbilical cord, and other tissues. They secrete anti‐inflammatory cytokines, reduce adipose inflammation, improve beta‐cell function, and, through immunomodulation, enhance insulin sensitivity in liver/muscles.	Early clinical trials for T2D and glucose tolerance. Some clinics offer IV infusions marketed for improved glycemic control.	[67, 68]
Adipose‐derived stem cells (ADSCs)	ADSCs, a subtype of MSCs isolated from adipose tissue, enhance IRS, improve the glucose uptake in skeletal muscles, and induce adipose tissue anti‐inflammatory effect.	Preclinical trials have rendered improved insulin sensitivity and reduced fasting glucose levels, following the ADSC administration.	[69]
Stem cell–derived beta‐like cells	Human‐induced pluripotent stem cells (hiPSCs) and human embryonic stem cells (hESCs) are differentiated into insulin‐producing beta‐like cells. Researchers are also designing “off‐the‐shelf” beta cells with distinctive surface proteins so as to provide protection by engineered immune cells.	FDA has approved the first donor islet cell therapy for T1D in 2023; T2D trials are ongoing.	[70]
Pancreatic islet cell transplantation	• Donor pancreatic islet cells from a deceased donor are infused, commonly into the liver, where they produce insulin independently.	FDA has approved the first donor‐derived pancreatic islet cell therapy for adults with Type 1 diabetes suffering from severe hypoglycemia. Clinical trials like the FORWARD study of stem cell–derived islet product VX‐880 showed reduced dependency on injectable insulin. Lantidra is approved for the treatment of adults with Type 1 diabetes and are unable to acquire the average blood glucose levels.	[71]
Porcine pancreatic islet xenotransplantation	Two immunosuppressive protocols are employed: one based on CD40‐CD40L blockade and another engaging clinically available immunosuppressants. Application presented a reduction in insulin dose and improved hypoglycemic awareness among the majority of subjects, with some patients achieving long‐term insulin independence.	Clinical trials have been conducted in Sweden, China, and Mexico, whereas microencapsulated neonatal porcine islet transplantation trials are in progress in New Zealand, Russia, and Argentina since 1994 till present.	[72]
CAR‐Treg and beta cell combo	It is a highly specific strategy to treat T1D. Chimeric antigen receptor (CAR)–modified engineered regulatory T cells (CAR‐Tregs) are combined with stem cell–derived beta cells to mobilize localized immune protection without systemic immunosuppression. The combo recovers insulin production and prevents autoimmune attack that exacerbates IR.	Preclinical phase at Medical University of South Carolina (MUSC) and the University of Florida (UF).	[73]

*(B) Subcellular Particle-Based Therapies*			
Extracellular vesicles (EVs)	Enlist nano‐ to micrometer vesicles that include exosomes and microvesicles. EVs are a combination of bioactive molecules (e.g., mRNAs, miRNAs, proteins, and lipids) that can be carried to the targeted cells/tissues *via* the blood or lymph circulation. MSC‐derived EVs offer a promising option for treating IR. EVs promote intercellular communication, reduce oxidative stress, and promote the glucose transporter (GLUT4) translocation and insulin signaling pathway modulation.	Cohort study: T2D patients on monthly IV + daily sublingual exosomes, resulting in discontinuation after 3 months. Ongoing clinical trials to determine their safety and clinical efficacy; still experimental.	[74, 75]
Mitochondrial transfer therapy	Mitochondrial dysfunction may accelerate the progression of insulin resistance and subsequent organ dysfunction by increasing reactive oxygen species production. Mechanisms of intercellular mitochondrial transfer include TNTs (transfer *via* tunneling nanotubes), extracellular vesicles (EVs), gap junction channels (GJCs), cell fusion, and mitochondrial extrusion. These therapies enhance ATP production, improve signaling and oxidative phosphorylation, and reduce reactive oxidative species (ROS).	1. Preclinical evidence: Mice HFD model: IV injection of MSC‐derived mitochondria improved glucose tolerance, reduced HOMA‐IR by 40% in a duration of 2 weeks. 2. Human myotubes: Mitochondrial transfer from healthy donors rescued palmitate‐induced IR *via* AMPK activation 3. Clinical evidence: T2D/IR: Phase I/II trials ongoing. 4. No FDA approval till date for the metabolic disease. Some private clinics offer this therapy.	[76]
Nanoparticle‐mediated RNA therapy	This therapy involves the delivery of therapeutic RNA molecules directly to insulin‐resistant tissues through nanocarriers. This therapy causes silencing of genes involved in inflammation, helps promote the insulin receptor signaling, and improves the glucose metabolism.	The complexity of reversing insulin resistance requires high‐precision delivery, due to the low stability and rapid degradation of naked RNA in the body. Further translational and safety studies are suggested prior to the successful transition of these nanoformulations from the laboratory into the formal Phase I and Phase II human trials.	[77]
Biomimetic nanovesicles	This novel biomimetic insulin delivery technique uses glucose‐responsive polymersome nanovesicle that is acid‐sensitive and undergoes the hydrolysis of assembled polymers and the dissociation of vesicles, thereby releasing the encapsulated insulin. In vivo studies demonstrated that this biocompatible nanovesicle is effective in regulating blood glucose levels for a longer period of time.	Preclinical research and development phase	[78, 79]
Oral glucose‐responsive nanoparticles	Oral glucose‐responsive nanoparticles are designed for pancreatic β‐cell regeneration and treatment of T2D. Carboxymethyl chitosan (CMC) grafted with 3‐aminophenylboronic acid (APBA) and molecule citrus pectin (MCP) spheres encapsulating artemisinin (Art) connected by borate ester bonds are used to prepare these nanoparticles. The CMC–APBA‐wrapped Art‐loaded MCP nanoparticles (CAM@Art) present therapeutic effects by improving drug retention and absorption in the intestine.	Preclinical stage	[80]
CRISPRa‐based Gene Activation Technique	CRISPR‐activated system targets the upregulation of endogenous genes (MAFA, NGN3, PDX1, IRS2, and FOXO1) that are involved in insulin sensitivity and β‐cell regeneration and are crucial regulators of pancreatic function and glucose homeostasis maintenance. The resultant improved metabolic regulation may contribute to β‐cell functional recovery and improve insulin signaling pathways.	Preclinical stage	[81]
Bioactive‐Based Nano Carriers	Biodegradable, biocompatible nanocarriers engineered to carry bioactive compounds in an encapsulated form for improved bioavailability and stability. These nanocarriers promote the pancreatic beta‐cell regeneration, improve glucose regulation, and reduce insulin resistance.	Preclinical stage	[82]
Proinsulin‐loaded NPs	Immunotherapy of T1D is carried out by these engineered entities through a dual‐action approach. The proinsulin fusion protein (autoantigen) and an immunomodulator ITE [2‐(1′H‐indole‐3′‐carbonyl)‐thiazole‐4‐carboxylic acid methyl ester] are used to create ultra‐tiny nanovehicles. ITE binds to and activates the aryl hydrocarbon receptor (AhR) pathway. “Tolerogenic” environment is created; hence, the immune cells’ invasion to the body’s healthy tissues stops. The NPs promote regulatory T cell’s (Treg) and regulatory B cell’s (Bregs) emergence. Dendritic cells are targeted, and their ability to activate autoreactive T cells is suppressed. Destruction of pancreatic β‐cells is discouraged without halting the entire immune system. Temporary or sometimes long‐term disease remission is achieved.	Preclinical stage	[83]
GLUT4/GLUT2 targeting particles	Novel pharmacological and natural dietary strategies employ neuropeptide (Spexin), plant alkaloids (Berberine), and other compounds as potential drug targets for clinically treating IR and hyperglycemia. This strategy exploits the glucose‐dependent metabolic reprogramming. Mechanism involves the obstruction of overactivation of certain glucose consumption genes, induced by turning off GAL2 receptors in myotubes or through the use of yeast *S. cerevisiae* GAL2 receptor antagonist. New role has therefore been assigned to the GAL2/GLUT4 signaling pathway in protecting against IR with Spexin. In addition to the GLUT4 combating IR *via* fixing of the IRS/Akt/GLUT4 signaling route, GLUT2 has also been identified as a way, how berberine derived from Coptidis rhizome transports PLC‐β2 to the membrane, thus reducing GLUT2 translocation and normalizing glucose metabolism.	Spexin: Early stages of pharmacological experimentation. Berberine: Available over the counter as a dietary supplement.	[84–86]

### 7.5. Prevention Strategies and Public Health Implications

i. Regulation of Sugar Consumption and Processed Food: Dietary patterns that are rich in ultraprocessed foods and added sugars significantly contribute to IR and metabolic disease. Policies such as front‐of‐pack labeling and taxation on sugar‐sweetened beverages (SSB) have shown measurable impacts. For instance, in 2021, after the implementation of Mexico’s soda tax, there was a 17% decrease in purchases of SSB, and national metabolic health surveillance revealed that due to this policy, there was an improvement in insulin and triglyceride levels of many individuals. Such regulatory tools are now included in the WHO’s recommended strategies for the prevention of diabetes [87].
ii. School and Workplace‐Based Health Promotion: Integrating nutrition reform, physical activity, and health education into institutional settings leads to the prevention of long‐term risk of diabetes. In the Sustain Health School study, a multiple‐component intervention comprising nutrition education, physical education, and restrictions on SSB led to a reduction in waist circumference and improved insulin sensitivity markers in adults [88]. Likewise, a meta‐analysis conducted in 2024 demonstrated that there was about a 26% improvement in insulin sensitivity across pooled corporate interventions involving workplace wellness programs aimed at sedentary employees [89].
iii. Maternal and Early Life Interventions: Prevention of IR begins even before birth. Gestational diabetes and maternal obesity increase the offspring’s lifetime risk of IR. Programs encouraging preconception health, exclusive breastfeeding, and optimal maternal weight gain have shown long‐term metabolic benefits. In 2021, a birth cohort study found that infants born to mothers who maintained optimal gestational glucose control had a 32% lower risk of juvenile IR by Age 68 [90].
iv. Mobile‐Based and Digital Health Interventions: With day‐by‐day increasing digital access, smartphone apps and telemedicine are becoming scalable tools for the prevention of diabetes. Programs such as mDiabetes used SMS‐based lifestyle coaching and reminders to reach over 1 million individuals, leading to a 20% decrease in progression from prediabetes to diabetes for over 18 months. These platforms provide low‐cost and personalized interventions that can be included in national prevention frameworks [91].
v. Urban Design and Policy to Encourage Physical Activity: Urban and environmental planning strategies, including bicycle infrastructure, walkable neighborhoods, and public green spaces, promote active lifestyles and reduction in obesity‐related IR. A longitudinal cohort study conducted in Copenhagen discovered that adults living in walkable neighborhoods were 40% less likely to develop IR, regardless of socioeconomic status. WHO and IDF now encourage policy‐driven urban planning that supports policy‐driven urban planning as a public health approach [92].
vi. Surgical interventions: Surgical procedures such as sleeve gastrectomy (SG) and Roux‐en‐Y gastric bypass (RYGB) not only induce substantial weight loss but also lead to marked insulin sensitivity [93]. These surgical interventions improve glucose homeostasis by modulating gut hormones (e.g., GLP‐1), by altering bile acid metabolism, and by reducing systemic inflammation. Clinical trials and longitudinal studies have shown that bariatric surgery can delay or prevent the onset of T2DM in individuals at high risk of IR [94].

### 7.6. Current Medical Guidelines for Prevention of IR

Recent medical guidelines emphasize early detection, risk assessment, and intervention for individuals who are at risk and already have IR, even before hyperglycemia develops. The World Health Organization highlights IR as an amenable risk factor in its Global Diabetes and Noncommunicable Diseases prevention strategy. WHO guidelines emphasize the primordial importance of preventing IR through national efforts, thereby targeting poor nutrition, obesity, and physical inactivity [95]. The International Diabetes Federation also considers IR as a pivotal risk factor for T2DM. IDF recommends the standardization of IR screening tools in both community and clinical settings to ensure timely intervention and diagnosis [96]. Notably, the National Institute for Health and Care Excellence (NICE) also updated its recommendation in 2022 to prioritize preventive measures for IR in adults who are obese [97].

## 8. Research Gaps and Future Directions

### 8.1. Correlation vs. Causality: Unresolved Issues

Although considerable evidence links IR to diabetes, the directionality of this is still not clear. The main gap lies in the lack of human longitudinal studies that can confirm whether IR is a consistent precursor to diabetes or an outcome of persistent metabolic dysfunction. This uncertainty is especially evident in T1DM, in which the emergence of IR is later due to factors such as insulin therapy and obesity, yet remains poorly studied. Furthermore, the field lacks a standard definition of IR, and surrogate markers such as HOMA‐IR differ across various populations, which restricts the capability to draw causal inferences [98].

Furthermore, while genetic studies indicate a common predisposition to IR and T2DM, the functional importance of many associated genes is still unidentified. Compensatory hyperinsulinemia is still understudied as a potential driver of IR rather than just a response [37]. To resolve these challenges, future research should focus on exceptional longitudinal studies, validation of novel molecular biomarkers, and integration of multiomics data. Epigenetic and genetic markers associated with insulin sensitivity need to be explored as causal predictors, not just associations [99]. Animal models that imitate both the development and reversal of IR should be further improved to test the causal hypothesis. Furthermore, standardizing diagnostic thresholds for IR across clinical studies is crucial for ensuring comparability and reproducibility. These effects are crucial for establishing a clear causal model and developing effective early interventions for the prevention of diabetes.

### 8.2. IR in Youth and Lean Individuals

IR in youth and lean individuals is a growing concern that is poorly understood. The majority of research related to IR has focused on mechanisms that are related to obesity, leaving a gap in understanding of how IR develops in normal body weight individuals. Chronic stress, ectopic fat disposition, genetic predisposition, and mitochondrial dysfunction are the factors that may lead to IR in slim individuals, but there is still a need for further research regarding these mechanisms. Similarly, rising cases of IR in youth are connected to early life exposures, pubertal IR, and sedentary behavior. However, data from long‐term progression from youth to adulthood are sparse [100]. Recent screening criteria based on BMI usually miss nonobese, unhealthy individuals who are at risk, thereby resulting in delayed diagnosis, so future guidelines must emphasize metabolic screening regardless of their body size. Future studies should look into lean and pediatric phenotypes of IR by using genetic profiling, advanced imaging, and longitudinal cohorts, as well as lean IR biomarkers such as adiponectin and ectopic intramyocellular or hepatic fat for improving targeted interventions and early detection beyond weight‐based models [101].

### 8.3. Role of Precision Medicine and Multiomics as Future Approaches in Prevention of IR

To unravel disease mechanisms variability in individuals, future strategies for the prevention of IR must shift toward multiomics approaches and precision medicine. These methods play an important part in the identification of genetic predispositions and early biomarkers that cause differential insulin responses, even among individuals with similar clinical profiles.

Integration of multiomics approaches may enable tailored prediction of risk and personalized treatments. Genomics can help in the identification of SNPs linked to defects in insulin signaling, whereas metabolomics can reveal profiles of amino acids and lipid intermediates that indicate subclinical IR [37]. Microbiome profiling has been revealing strong correlations between impaired insulin sensitivity and gut dysbiosis, indicating opportunities for therapies that are microbiota‐targeted [102].

Platforms of precision medicine will also enable patient stratification for targeted therapeutic strategies, such as responsiveness to personalized dietary plans or responsiveness to metformin. Furthermore, single‐cell transcriptomics will further reveal how different cell types within adipose tissue and the pancreas lead to the progression of IR. Despite promising research, future research must prioritize the validation of these biomarkers across diverse populations and translate omics discoveries into clinical‐grade, accessible diagnostic tools [103]. Building huge multiethnic biobanks and incorporating artificial intelligence for the interpretation of data will be vital for realizing the precision medicine’s full potential for combating IR [104].

## 9. Conclusion

This comprehensive review underscores the complex and bidirectional relationship between IR and DM, thereby emphasizing the fact that IR can act as both cause and consequence of disease progression in both T1DM and T2DM. Therapeutic hyperinsulinemia and chronic hyperglycemia aggravate IR via AGEs, altered adipokine profiles, and proinflammatory signaling. Moreover, the presence of IR in youth and nonobese individuals emphasizes the prevailing diagnostic models that are weight‐centric. Experimental models and epidemiological data confirm the self‐perpetuating cycle between IR and hyperglycemia, reinforcing the need for early detection of IR and hyperglycemia by using validated biomarkers and techniques. The existence of IR even in nonobese and youth populations highlights the limitations of weight‐centric diagnostic paradigms, thereby emphasizing the need for more inclusive treatments. Lifestyle modifications, pharmacologic treatments such as SGLT2 inhibitors and metformin, and public health policies targeting early life exposures and dietary patterns demonstrate potential in halting the progression of IR. In conclusion, this review highlights key research gaps, particularly the need for causal human investigations, characterization of lean phenotypes, and precision medicine using multiomic approaches to tailor prevention and treatment strategies. These findings reinforce that breaking the vicious cycle of IR and diabetes at multiple mechanistic, clinical, and societal levels is crucial for curing the worldwide diabetes epidemic.

## Author Contributions

Shafa Shahid and Saadia Qamar contributed to the literature search, data extraction, and drafting of the manuscript. Fouzia Qamar assisted in organizing and synthesizing the literature and contributed to critical revisions. Shafa Shahid originally created the figures and tables. Aliza Fatima refined the figures and tables in BioRender (licensed version), made graphical amendments to improve visual clarity, and also assisted in the compilation of references. Muniba Khaliq conceptualized and supervised the review, provided critical guidance throughout the writing process, and is the corresponding author.

## Funding

No funding was received to assist with the preparation of this manuscript.

## Disclosure

All authors read and approved the final version of the manuscript.

## Conflicts of Interest

The authors declare no conflicts of interest.

## Data Availability

The authors have nothing to report.
